# Northern Ohio Dental Association

**Published:** 1892-06

**Authors:** 


### Northern Ohio Dental Association.

The Thirty-Third Annual Meeting of the Northern Ohio Dental Association was held in Cleveland, Ohio, May 10th, 11th and 12th, with an unusually large attendance of members and visitors. The programme as published in the *Register* was fully carried out, and all papers were read, with only one exception. Fourteen new members were added to the list of membership. The essays read at this meeting will appear in the *Register*. The next meeting will be held in Akron, May, 1893.

The list of officers for the following year is as follows:

President, W. H Whitslar, Cleveland, O; Vice President, S. B. Dewey, Cleveland, O.; Corresponding Secretary, Henry Barnes, Cleveland, O.; Recording Secretary, L. P. Bethel, Kent, O.; Treasurer, Charles Buffett, Cleveland, O. Executive Committee—Henry Barnes, J. R. Owens, (Cleveland,) F. Knowlton, (Akron), Grant Mitchell, (Canton). Membership Committee—Charles Buffett, (Cleveland), W. W. Fowler, (Painesville), S. W. Barker, (Toledo), C. Peck, (Sandusky).

A full and comprehensive programme will be announced soon for the next meeting. It has been made a rule of this society, that essayists shall send a copy of their essays to those who open the discussions, not less than thirty days previous to the meeting.

